# MicroRNAs Modulate Drug Resistance-Related Mechanisms in Hepatocellular Carcinoma

**DOI:** 10.3389/fonc.2020.00920

**Published:** 2020-06-30

**Authors:** Yuehui Liang, Qi Liang, Liang Qiao, Fang Xiao

**Affiliations:** ^1^Department of Health Toxicology, Xiangya School of Public Health, Central South University, Changsha, China; ^2^Department of Radiology, The Third Xiangya Hospital, Central South University, Changsha, China; ^3^Storr Liver Center, Westmead Institute for Medical Research, University of Sydney and Westmead Hospital, Westmead, NSW, Australia

**Keywords:** microRNAs, drug resistance, hepatocellular carcinoma, autophagy, tumor microenvironment

## Abstract

Primary liver cancer [hepatocellular carcinoma (HCC)] is one of the most common malignant tumors worldwide, causing serious health threats because of its high morbidity and mortality, rapid growth, and strong invasiveness. Patients with HCC frequently develop resistance to the current chemotherapeutic drugs, and this is largely attributed to the high-level heterogeneity of the tumor tissue. MicroRNAs (miRNAs) are a group of master regulators for multiple physiological and pathological processes and play important roles in the tumorigenesis. More recent studies have indicated that miRNAs also play a non-negligible role in the development of drug resistance in liver cancer. In this review, we summarize the data from the latest studies on the mechanisms of drug resistance in liver cancer, including autophagy, membrane transporters, epithelial–mesenchymal transitions (EMTs), tumor microenvironment, and genes and proteins that are associated with apoptosis. The data herein will provide valuable information for the development of novel approaches to tackle drug resistance in the management of liver cancer.

## Introduction

Primary liver cancer is one of the most common malignant tumors in the worldwide and is a serious health threat to humans because of its high morbidity and mortality, rapid growth, and strong invasiveness (1). According to the global cancer statistics, liver cancer ranks the second leading cause of cancer-related death, and hepatocellular carcinoma (HCC) accounts for 70–90% of primary liver cancer (2). About 850,000 new cases of HCC are diagnosed worldwide every year, and the number is still on the rise (3). Clinically, surgical resection is the first choice for the treatment of early primary liver cancer, but this treatment approach carries high recurrence rate and high rate of postsurgery metastasis, because most HCC patients are generally diagnosed at the advanced stage, and only palliative treatments such as chemotherapy can be used for treatment. Although chemotherapy may help delay or avoid tumor recurrence in the short term, its long-term role in extending patient survival is very limited (4).

It is well-known that HCC can rapidly develop resistance to the currently available chemotherapeutic drugs (5, 6). In the management of cancer patients, drug resistance is a critical hurdle for successful treatment. It has been reported that resistance to chemotherapeutic drugs accounts for more than 90% of cancer specific mortality (7). Based on the characteristics of cancer cell resistance, anticancer drug resistance can be divided into two categories: primary drug resistance (PDR) and multidrug resistance (MDR). In PDR, cancer cells may become resistant to one type of anticancer drugs but can still be sensitive to other anticancer drugs of different categories. In the MDR, cancer cells become resistant to the anticancer drugs, with different structures and mechanisms. MDR is the root cause of chemotherapy failure of HCC. Cancers with MDR exhibit several distinctive features as compared with those without. For example, cancers that developed MDR show a high level of apoptotic threshold, aerobic glycolysis, hypoxia, and elevated activity of drug-efflux transporters (8). These features render the tumor cells refractory to chemotherapy. The comparison of characteristics of resistant and non-resistant cancer cells is summarized in Table 1.

**Table 1:** The key characteristics of resistant and nonresistant cancer cells.

**Resistant cells**	**Nonresistant cells**
**Cellular level**
High G0 cell ratio	Low G0 cell ratio
More proliferative cell	More ready to undergo apoptosis
More mesenchymal feature	More epithelial feature
Multidirectional differentiation	No differentiation
Altered physiology	Physiology maintained
**Molecular level**
Alternative signaling pathway	Stable drug target
Activated drug efflux transporter	Inactivated drug efflux transporter
DNA repair enhancement	No or little DNA repair
High apoptosis threshold	Normal apoptosis threshold
Changed drug metabolism	Unchanged drug metabolism
Epigenetic change	Homeostatic responses to adversity

## MicroRNAs Modulate Drug Resistance-Related Mechanisms in Hepatocellular Carcinoma

MicroRNAs (miRNAs) are a series of short non-coding single-stranded RNA molecules encoded by endogenous genes with the molecular length of between 19 and 24 nt. They act mainly on the non-coding region at the 3′ end of the messenger RNA to degrade the target RNA or terminate the translation (9). A single miRNA can target several different mRNAs, and a single mRNA can be synergistically inhibited by a number of different miRNAs (10), thereby achieving miRNA-guided posttranscriptional regulation of the gene expression. Numerous studies have consistently proved that the occurrence and progression of HCC are closely related to the differential expression of miRNAs, and a considerable number of miRNAs are directly involved in the drug resistance of cancer cells (7, 11, 12). According to different regulatory mechanisms, miRNAs can promote or inhibit drug resistance of hepatoma cells. All the researches into the molecules involved in chemotherapy provide a deep understanding of the underlying mechanism of drug resistance. Understanding the mechanisms of how miRNAs are involved in the development of drug resistance can facilitate the development of novel approaches to tackling drug resistance in cancer therapy. Below, we will summarize the data from the latest studies on how HCC develops resistance to chemotherapies (13–17).

### Autophagy

Autophagy is a continuous metabolic process that maintains homeostasis and normal cellular function by degrading and restoring damaged organelles, long-lived, and misfolded proteins in cells (18). The signals that activate the autophagy process usually come from different stress states, such as starvation, hypoxia, oxidative stress, and protein aggregation. The formation of the autophagosome relates to the evolutionarily conserved genes, autophagy-related genes (ATGs), and is usually divided into different stages: initiation, nucleation of the phagophore, formation of autophagosome membrane, fusion with the lysosome, and the degradation of products in vesicles (19). The initiation begins with the activation of the common targets unc-51-like autophagy activating kinase 1 (ULK1) (also known as ATG1) complex (20, 21). In the expansion stage, ATG12–ATG5 binds to the ATG16 complex to enlarge the autophagosome membrane and promotes the lipidation of microtubule-associated protein1 light chain3 (MAP1LC3; also known as LC3). In addition, ATG4B binds to ATG7, allowing LC3I and phosphatidylethanolamine (PE) to combine to form LC3II, a lipid-conjugated form of LC3 that commonly considered as a biomarker for autophagosomes (22–24). Ultimately, autophagosomes fuse with lysosomes to form autophagy lysosomes. At present, the role of autophagy in the resistance of HCC remains controversial. Autophagy occurs frequently during tumorigenesis and cancer chemotherapy. Autophagy pathways have been reported to be involved in the development of MDR (23). Thus, anticancer drugs that can directly induce autophagy of MDR cancer cells would be of great therapeutic value. Autophagy can act as a housekeeper to clear damaged organelles or other cellular components and thus protect other cells. Excessive or sustained autophagy has been shown to promote cancer cell death by enhancing the process of apoptotic induction or triggering “autophagic cell death,” which is different from type I programmed cell death (apoptosis) (23). Autophagy is closely associated with the formation and progression of cancer and MDR in the clinical settings. Tumors at the advanced stage generally express a higher level of the ATGs (e.g., mTOR, Raptor, Rictor, and LC3) than do the tumors of the early stage, and the expression level of ABCB1(MDR1) in the tumors that developed autophagy is positively correlated with the level of LC3 and Beclin1 (25). On the one hand, autophagy mediates the acquired resistance phenotype of cancer cells during chemotherapy. Hence, inhibition of autophagy can sensitize drug-resistant cancer cells to chemotherapeutic agents. However, autophagy may also induce autophagic cell death. Thus, if applied properly, autophagy can be used to improve the therapeutic effect of MDR cancer, and the role of autophagy in MDR must be elucidated.

In sum, based on the published literatures, autophagy can promote drug resistance in most cases, and miRNAs can make HCC cells resistant or sensitive to anticancer drugs by positive or negative regulating autophagy.

#### MicroRNAs Make Hepatocellular Carcinoma Cells Resistant to Drugs by Positive Regulating Autophagy

There is abundant evidence that MDR codevelops with autophagy and miRNAs. The coexistence of autophagy and miRNAs in patients with poor prognosis indicates that both autophagy and miRNA may promote the occurrence of MDR. MiR-185 is an attractant for autophagy. However, when autophagy is inhibited, miR-185 mimics induce more apoptosis (26). It is suggested that miRNAs can positively regulate autophagy and participate in tumor cell resistance. A similar study also has found that overexpression of miR-494 in HCC cells activated by the mTOR pathway increases the resistance of sorafenib (27). Histone deacetylase inhibitors (HDACis) are a group of drugs recommended for the treatment of many solid tumors. Cancer cells resistant to HDACis express high levels of the transcription factor nuclear factor erythroid 2 like-2 (Nrf2), and autophagy has been shown to contribute to Nrf2-mediated drug resistance (28). Treatment of cancer cells with HDACi leads to increased Nrf2 expression via upregulation of miR-129-3p. In HDACi-induced autophagy, Nrf2 positively regulates mTOR through miR-129-3p, and HDACi-mediated cell death can be enhanced by inhibiting miRNA-129-3p (28). The role of autophagy in drug resistance is also supported by a recently published study where targeting the PU.1-miR-142-3p-ATG5/ATG16L1 axis can inhibit the cyto-protective autophagy and overcome sorafenib resistance (29). In the 5-fluorouracil (5-FU; a widely used antineoplastic drug) treated cells, the expression levels of miR-216b and lncRNA MALAT1 are twice as high as those in the control group. Yuan et al. (30) have found that the mechanism may be related to autophagy caused by a high expression of LC3-II and downregulation of p62 in drug-resistant cells, and MALAT1-miR-216b axis modulates MDR of HCC cells by participating in the regulation of autophagy.

#### MicroRNAs Make Hepatocellular Carcinoma Cells Sensitive to Drugs by Negatively Regulating Autophagy

Recent studies have reported that autophagy induced by chemotherapeutics may promote resistance of cancer cells to drugs. MiRNAs can induce autophagy negatively in the process of drug resistance. Jin et al. (31) have found that doxorubicin (Dox) could induce autophagy and reduce the level of miR-26a/b in HCC cells. Conversely, when the expression of miR-26a/b maintains at the physiological level, Dox-induced autophagy has been inhibited and increased apoptosis in HCC cells. Furthermore, overexpression of miR-26a/b increases the sensitivity of HCC cells to Dox through negatively regulating ULK1 and promoting apoptosis. MiR-101 can also enhance chemosensitivity of HCC cells and promote drug-induced apoptosis through inhibiting autophagy (32). Involvement of autophagy and miRNAs in the development drug resistance is likely a universal mechanism. For example, cisplatin-based treatment significantly reduces miR-199a-5p expression but activates autophagy (33). On the other hand, overexpression of miR-199a-5p enhances the inhibitory effect of cisplatin on cell proliferation. Similarly, blockage of autophagy also inhibits cell proliferation induced by downregulation of miR-199a-5p, suggesting that miR-199a-5p regulates chemoresistance through autophagy.

In addition to acting on autophagy individually, miRNAs can also bind to other carrier substances and inhibit other key molecules of autophagy to enhance drug sensitivity. For example, autophagy has been proved to promote sorafenib resistance, and miR-375 has been indicated to inhibit autophagy (34). Further research has found that miR-375/Sf-LCC NP can transport sorafenib and miR-375 into HCC cells and tumor tissues, thereby increasing the retention time of sorafenib, inhibiting autophagy, and producing enhanced anti-tumor effects (35). A study reported by Gao et al. (36) indicated that a dietary flavonoid apigenin significantly enhances Dox sensitivity and miR-520b expression in HCC. Furthermore, miR-520b can inhibit ATG7, an important autophagy-dependent molecule, indicating that the miR-520b/ATG7 axis has a positive effect on the chemosensitivity of HCC.

**Figure 1:**
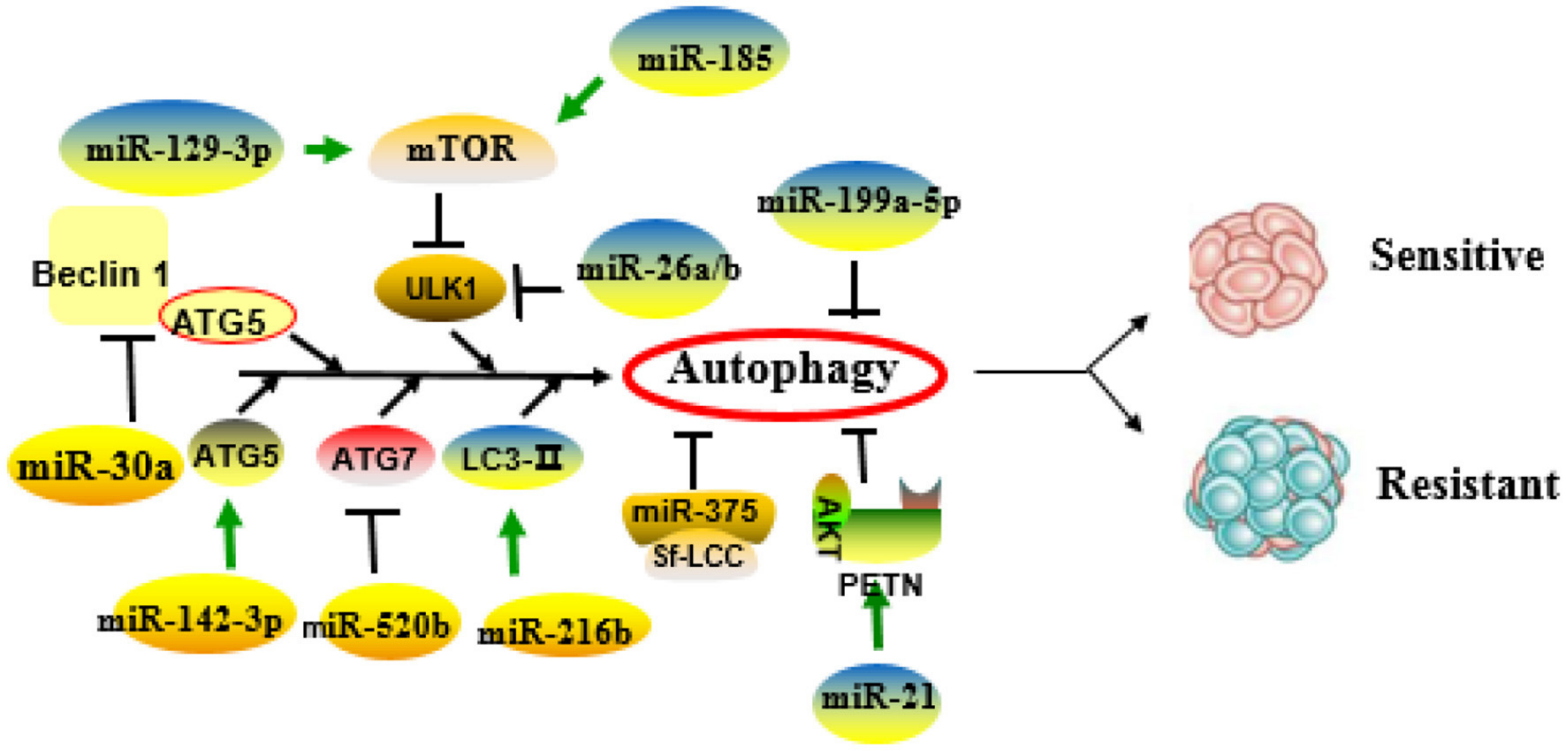
Role of microRNAs (miRNAs) in the regulation of autophagy-related drug resistance. MiRNAs exert important roles in drug resistance through positive or negative regulation of key molecules or pathways of autophagy.

Figure 1 and Table 2 summarize the role of miRNAs in the regulation of autophagy-mediated drug resistance.

**Table 2:** MiRNAs known to modulate chemoresistance through regulating autophagy.

**MiRNA**	**Target**	**MiRNA regulated autophagy**	**Autophagy regulated resistance**	**Drug**	**Reference**
MiR-185		Positively	Positively		(26)
MiR-494	mTOR	Positively	Positively	Sorafenib	(27)
MiR-129-3p	Nrf2, mTOR	Positively	Positively	HDACis	(28)
MiR-142-3p	ATG5/ATG16L1	Positively	Positively	Sorafenib	(29)
MiR-26b	LC3-II, P62	Positively	Positively	5-FU	(30)
MiR-26a/b	ULK1	Negatively	Positively	Doxorubicin	(31)
MiR-101		Negatively	Positively	Cisplatin	(32)
MiR-199a-5p		Negatively	Positively	Cisplatin	(33)
MiR-375		Negatively	Positively	Sorafenib	(35)
MiR-520b	ATG7	Negatively	Positively	Doxorubicin	(36)
MiR-30a	Beclin1, ATG5	Negatively	Positively	Sorafenib	(37)

*miRNAs, microRNAs; HDACis, histone deacetylase inhibitors; 5-FU, 5-fluorouracil*.

However, miRNAs can also inhibit autophagy through interacting with other pathways to achieve drug resistance. MiR-21 is proved to be upregulated in sorafenib-resistant HCC cells (38). Moreover, miR-21 was shown to inhibit the expression of phosphatase and tensin homolog deleted on chromosome 10 (PTEN) and to activate AKT pathways (39). He et al. have found that the activated AKT/PTEN pathway in sorafenib-resistant HCC cells exerts a positive effect on sorafenib resistance (40). The resistance to sorafenib can be restored when the HCC cells are treated with miR-21 mimics, and this effect is likely mediated through inhibiting autophagy *via* the AKT/PTEN pathway. Tumor metastasis is one of the adverse outcomes in patients with chemoresistance. MiR-30a is previously reported to be associated with vascular invasion, metastatic potential, and HCC recurrence (37). Fu et al. have reported that downregulation of miR-30a mediates Beclin1 and ATG5-dependent autophagy in metastatic HCC, resulting in “anoikis resistance,” which is considered to be the first step in metastasis of HCC cells (41).

### Membrane Transporter

MDR in HCC cells is mainly associated with two classical families of membrane transporters: (1) ATP-binding cassette (ABC) transporter superfamily, including P-glycoprotein (P-gp; also known as MDR1/ABCB1), MDR-associated protein1 (MRP1)/ABCCl, and breast cancer resistance protein (BCRP)/ABCG2. These transporters function as energy-dependent transmembrane drug delivery pumps and can pump intracellular substances, including a variety of anticancer drugs, out of cancer cells, leading to chemoresistance and chemotherapy failure; (2) solute carrier (SLC) transporter superfamily. Transporters in this category can disrupt the absorption of anticancer drugs in cells. Overexpression of these two types of transporters can induce drug resistance in cancer cells, but the ABC-transporter proteins are the main cause of drug resistance in hepatoma cells (42–44).

#### P-glycoprotein/MDR1/ABCB1

P-gp is a transmembrane glycoprotein encoded by MDR gene MDR1 with a molecular weight of ~170 kDa. It exhibits a high expression on multidrug-resistant cell membranes and can pump out anticancer drugs (45), resulting in the decrease in intracellular drug concentration, reduced drug cytotoxicity, and ultimately drug resistance. In addition, P-gp has recently been shown that it may confer resistance to chemotherapy-induced apoptosis (46). P-gp has a very broad spectrum of substrates that can export various drugs out of cells (47). The high level of P-gp in drug-resistant cells may be due to two mechanisms: a high basal level of P-gp in cancer tissue and chemotherapy-induced high expression in cancer cells (48). Previous studies have reported that the expression of miRNAs in drug-resistant cells of various tissues and organs was negatively associated with the expression of ABCB1 (49, 50), suggesting that the expression of some miRNAs in HCC was likely related to the alterations of MDR-1/P-gp expression and change of MDR phenotype. To support this notion, high expression of miR-122 in HCC cells resulted in a decrease in the expression of MDR-related genes, including P-gp (48). In another study, Yang et al. (51) have found that overexpression of miR-223 enhanced the sensitivity of HCC cells to anticancer drugs, whereas overexpression or silencing ABCB1 in these cells could rescue the response to anticancer drugs. Another miRNA is miR-27a, which has been reported to promote the occurrence and differentiation of various tumors (52). Moreover, miR-27a can also act as a chemotherapeutic drug resistance gene in multiple cancers including ovarian cancer, leukemia, pancreatic cancer, and adenocarcinoma. Chen et al. (53) have demonstrated that overexpression of miR-27a in the BEL/5-FU cells (multidrug-resistant cell line of liver cancer) reduced the expressions of MDR1/P-gp and β-catenin but enhanced the sensitivity of these cells to 5-FU-induced apoptosis, suggesting that miR-27a may be a new therapeutic target for HCC.

#### MRP/ABCC

MRP, also known as ABCC, mediates the efflux of potentially harmful substances in cells. Among them, ABCC1, ABCC2, and ABCC3 can participate in the MDR of malignant tumors, but their ability to induce drug resistance is lower than that of ABCB1. ABCC1 and ABCC2 cannot transport unmodified foreign substances (54). Exogenous substances must first bind to glutathione (GSH) under the action of glutathione thiotransferase (GST) and then be transported out of the cells through ABCCl and ABCC2 (55, 56). At present, there are few reports on miRNA regulation of MRP against the uptake and translocation of anticancer drugs. A study reported by Ma et al. (57) have shown that ABCCI 3′-untranslated region (3′-UTR) has binding sites for miR-133a and miR-326. Both miR-133a and miR-326 can reduce the expression of ABCCl in the hepatoma cell line HepG2 and enhance the sensitivity of the cells to Dox, suggesting that miR-133a and miR-326 mediate MDR through modulating ABCC1. However, as MRP mRNA may have a different secondary structure, the miRNAs may produce different biological functions when they act on different MRPs. For example, miR-379 downregulates ABCC2 in a haplotype-dependent manner, in that ABCC2 can be slightly inhibited when the haplotypes are wild type, whereas ABCC2 can be more strongly inhibited when the haplotypes are mutant (58).

**Figure 2:**
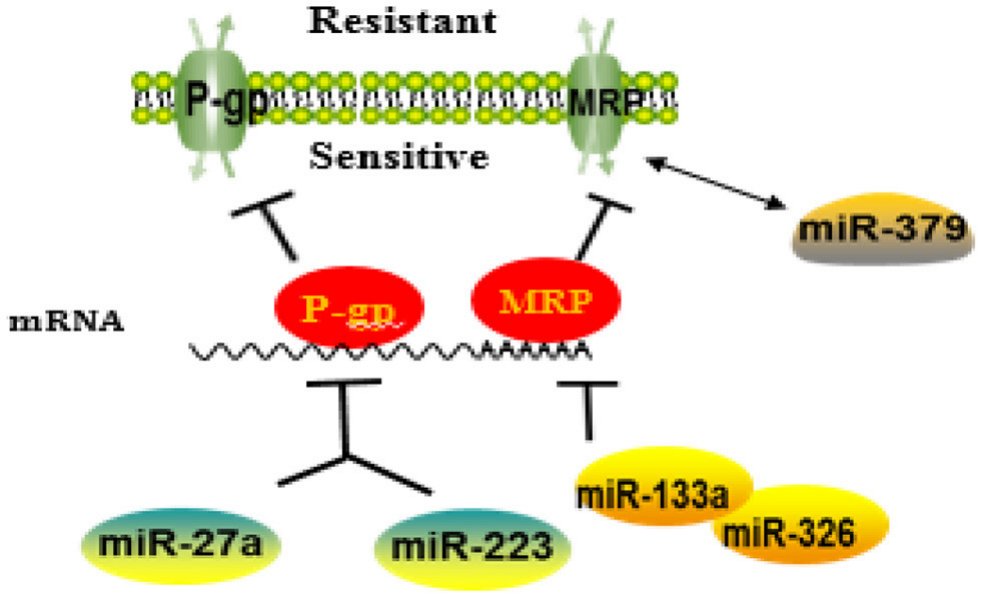
A schematic diagram illustrating the mechanisms of how microRNAs (miRNAs) regulate membrane transporter in the development of drug resistance. MiRNAs may affect the entrance of chemotherapy drugs into cells by negatively regulating membrane transport receptor.

MiRNAs Involved in Membrane Transporter-Mediated Resistance Are Summarized as Figure 2.

### Epithelial–Mesenchymal Transition

Epithelial–mesenchymal transition (EMT) is the process of transforming closely connected and immobile epithelial cells into loosely connected and migrating stromal tissue (59). The reversal process is called mesenchymal–epithelial transition (MET). In order to achieve phenotypic transformation, cells need to reprogram their gene expression (60). Reprogramming of EMT-related gene sequences means that E-cadherin downregulation leads to the separation of cell junctions, thereby promoting the transformation of epithelial phenotype to mesenchymal phenotype and the alteration of genes that encode cytoskeleton (61). Currently, three well-established groups of transcriptional regulators have been identified as important factors regulating the expression of EMT markers, including snail zinc finger protein family (Snail1 and Snail2); zinc finger E-box binding family (Zeb1 and Zeb2); and basic helix-loop-helix family (Twist1, Twist2, E12, E47, and differentiation inhibitory proteins). These transcription factors can independently or synergistically inhibit the expression of epithelial genes and activate interstitial genes, thereby regulating the EMT (62, 63).

By undergoing EMT, cancer cells can acquire resistance to chemotherapies (15). The relationship between EMT and drug resistance was first discovered in 2008 and was further verified by the findings that blocking or reversing EMT could convert the resistant cells into sensitive cells (64). In addition, the role of EMT in drug resistance has been demonstrated by the studies showing that anticancer therapies could render the cancer cells to acquire cancer stem cell (CSC) characteristics through EMT (65). It is now known that many signaling pathways, including TGF-β, AKT, ERK, Notch, and Wnt signaling, affect the chemoresistance of HCC cells by participating in the EMT process (66, 67). Recently, multiple miRNAs have been indicated to play key roles in the key regulation of EMT-related cancer resistance. For example, resistance against anti-EGFR therapies (e.g., cetuximab, gefitinib, and erlotinib) has been demonstrated to be closely related to the acquisition of EMT phenotypes *via* loss of miR-200c in a variety of cancer types (68). MiRNA-200 family (including miR-200a, miR-200b, miR-200c, miR-141, and miR-429) directly target and regulate the expression of the E-cadherin transcriptional repressors Zeb1 and Zeb2 in multiple cancers, and the inhibition of miR-200c increases E-cadherin expression and induces EMT (69). Another miRNA miR-130a-3p has been reported to regulate gemcitabine resistance. It has found that miR-130a-3p could target Smad4 and that overexpression of miR-130a-3p or downregulation of Smad4 could inhibit cell detachment, adhesion, migration, and invasion of gemcitabine-resistant HCC (GR-HCC) cells (70). Platelet-derived growth factor-D (PDGF-D), a critical regulator for EMT, has been reported to regulate gemcitabine resistance (71). PDGF-D is highly expressed in GR-HCC cells and can significantly inhibit miR-106a expression and subsequently promote Twist1 overexpression *in vitro*. Finally, the miRNA miR-122 has been reported to play an important role in HCC drug resistance through regulating EMT. It has been shown that overexpression of miR-122 could significantly inhibit EMT through repressing the level of Zeb1/2, Snail1/2, N-cadherin, and vimentin but upregulating E-cadherin expression. On the other hand, downregulation of miR-122 induces the opposite effect (72). Xia et al. have demonstrated that miR-153 inhibited Snail by directly targeting its 3′-UTR (73). In addition, studies on miRNAs have shown that miRNAs responded to the regulation of tumor EMT pathway genes. MiR-195 has been proven to inhibit Smad7 expression by binding to its 3′-UTR, thereby enhancing TGF-β-Smad signaling (74). MiR-122 represses MDR1 expression *via* targeting the Wnt/β-catenin pathway directly, strengthening the sensitivity of HCC to oxaliplatin (75). Immunotherapy stimulates the body's specific immune response by enhancing the self-regulating ability of the immune system and playing a positive role in improving the prognosis. Zeb1 is reported to relieve miR-200 repression of PD-L1 on cancer cells, leading to CD8^+^ T-cell immunosuppression and metastasis (76).

**Figure 3:**
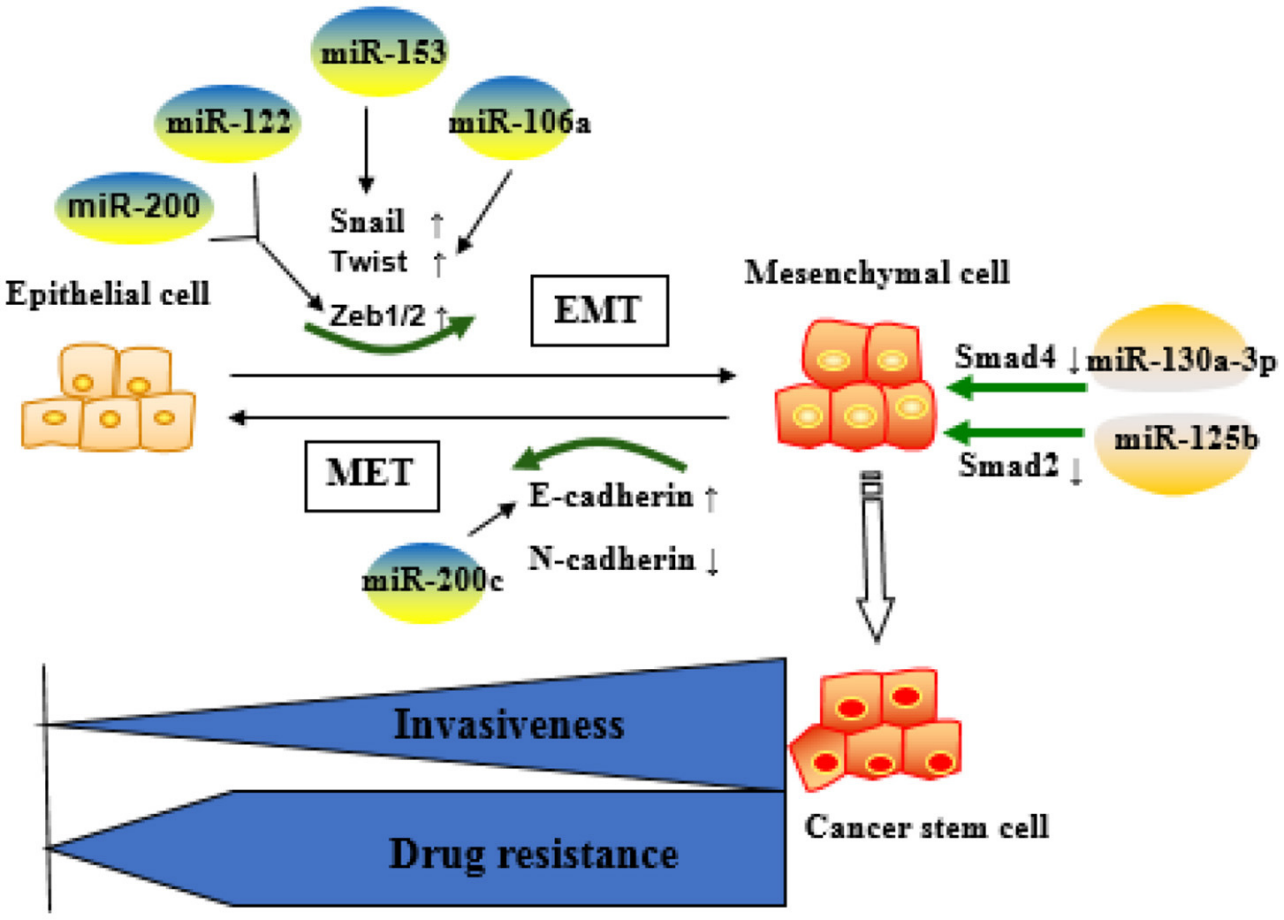
Role of microRNAs (miRNA)-regulated epithelial–mesenchymal transition (EMT) in the development of resistance. Epithelial cells are more sensitive to chemotherapeutic drugs, whereas mesenchymal cells are resistant to the drugs and more aggressive. MiRNAs mainly affect drug resistance through negative regulation of key molecules in the EMT process.

These reports indicate that the miRNAs are key factors in determining the fate of drug-resistant cancer cells by regulating EMT. Commonly identified miRNAs involved in EMT-mediated resistance are summarized as Figure 3.

### Tumor Microenvironment

The hepatic tumor microenvironment is a special environment for tumor cell growth formed by the interaction between tumor cells and extracellular matrix (ECM) (77). The main components of tumor microenvironment include (a) cellular components, such as angiogenic cells, immune cells, tumor-associated fibroblasts, endothelial cells, and adipocytes; (b) physical components in the ECMs; and (c) biochemical components, including cytokines, adhesion molecules, and oxygen tension (78, 79). Drug resistance occurs not only in cancer cells but also in the microenvironment where cancer cells reside. The microenvironment-mediated MDR is a result of continuous crosstalk between the cancer cells and their surrounding stroma. Soluble factors secreted by tumor cells or stromal cells can induce microenvironment-mediated drug resistance and subsequently favor tumor progression (80). Adhesion of tumor cells to stromal fibroblasts or to ECM components can also blunt therapeutic response (77, 78).

#### Cancer Stem Cells

CSCs are defined as a small proportion of cancer cells with strong self-renewal capacity and multidirectional differentiation potential (81). Most CSCs are in the G0 phase of cell cycle and are resistant to chemotherapy and radiotherapy, leading to tumor relapse and treatment failure (82, 83). Conventional therapies often fail because they do not completely eradicate CSCs, as this subset of cells often evades the treatment via therapy-induced EMT. Importantly, non-CSCs can spontaneously undergo EMT-like changes in response to chemotherapy and acquire CSC-like features, thereby leading to drug resistance (65).

CSCs have been identified in many types of cancers, such as liver cancer, colorectal cancer, pancreatic cancer, lung cancer, and breast cancer (84). Liver cancer stem cells (LCSCs) are now considered the source cells for the initiation, progression, and recurrence of liver cancer (85, 86). Recent studies have shown that that a variety of miRNAs can regulate the MDR of LCSCs through interacting with key molecular pathways such as Wnt/β-catenin, TGF-β, and JAK/STAT signaling (86). For example, Let-7 miRNAs, especially let-7a, can eliminate LCSCs via inhibiting Wnt signaling pathway, thereby significantly improving the sensitivity to chemotherapeutic agents (87). Tumor necrosis factor (TNF)-associated apoptosis-inducing ligand (TRAIL) is considered as a promising anticancer molecule, as it can selectively induce apoptosis in cancer cells without damaging normal cells. MiR-25 is significantly upregulated in LCSCs as compared with the non-CSCs, and the resistance of CSC to TRAIL appears to be mediated by miR-25, because knockdown of miR-25 significantly increases the sensitivity of LCSCs to TRAIL-induced apoptosis *via* PTEN/PI3K/AKT/Bad signaling pathway (88). Furthermore, downregulated miR-148b maintains CSC properties by targeting NRP1 in HCC (89). In another example, miR-125b has been found to be negatively correlated with EMT phenotype and CSC marker expression in HCC specimens, and consequently, overexpression of miR-125b could attenuate the EMT phenotype in HCC cancer cells by targeting Smad2 and Smad4 (90).

#### Hypoxia and the Warburg Effect

Rapid tumor growth increases the diffusion distance and oxygen consumption. Meanwhile, abnormal proliferation of tumor vasculatures and collapse of the blood vessel wall may impair the balance between the oxygen supply and demand of tumor cells, leading to relative hypoxia in the tumor environment. The survival of tumor cells in the hypoxic regions and the progression of solid tumors are regulated by hypoxia-inducible factor (HIF) (91). HIF is a heterodimer composed of an α subunit and a β subunit, mainly containing HIF-1α, HIF-2α, and HIF-β (92). It is known that HIF can interact with miRNAs in the regulation of drug resistance (93). For example, miR-338-3p has been found to enhance the sensitivity of HCC cells to sorafenib and promote HCC apoptosis by downregulating HIF-1α (94). HIF-1a can also achieve drug resistance by maintaining the characteristics of CSC. MiR-16 has been shown to reverse sorafenib resistance in HCC cells through targeting 14-3-3eta 3′-UTR and inhibiting 14-3-3eta/HIF-1α/CSC (95). Similar studies have been reported on the correlation between lncRNA MALAT1 and HIF α subunits, in HCC cells, confirming at least two miR-216b binding sites in lncRNA MALAT1, suggesting a possible role of HIF-2α-MALAT1-miR-216b axis in the regulation of MDR in HCC (30). During immunotherapy, hypoxia-induced miR-210 can significantly reduce the sensitivity of tumor cells to cytotoxic T lymphocyte (CTL)-mediated lysis via targeting PTPN1, HOXA1, and TP53I11 (96).

**Figure 4:**
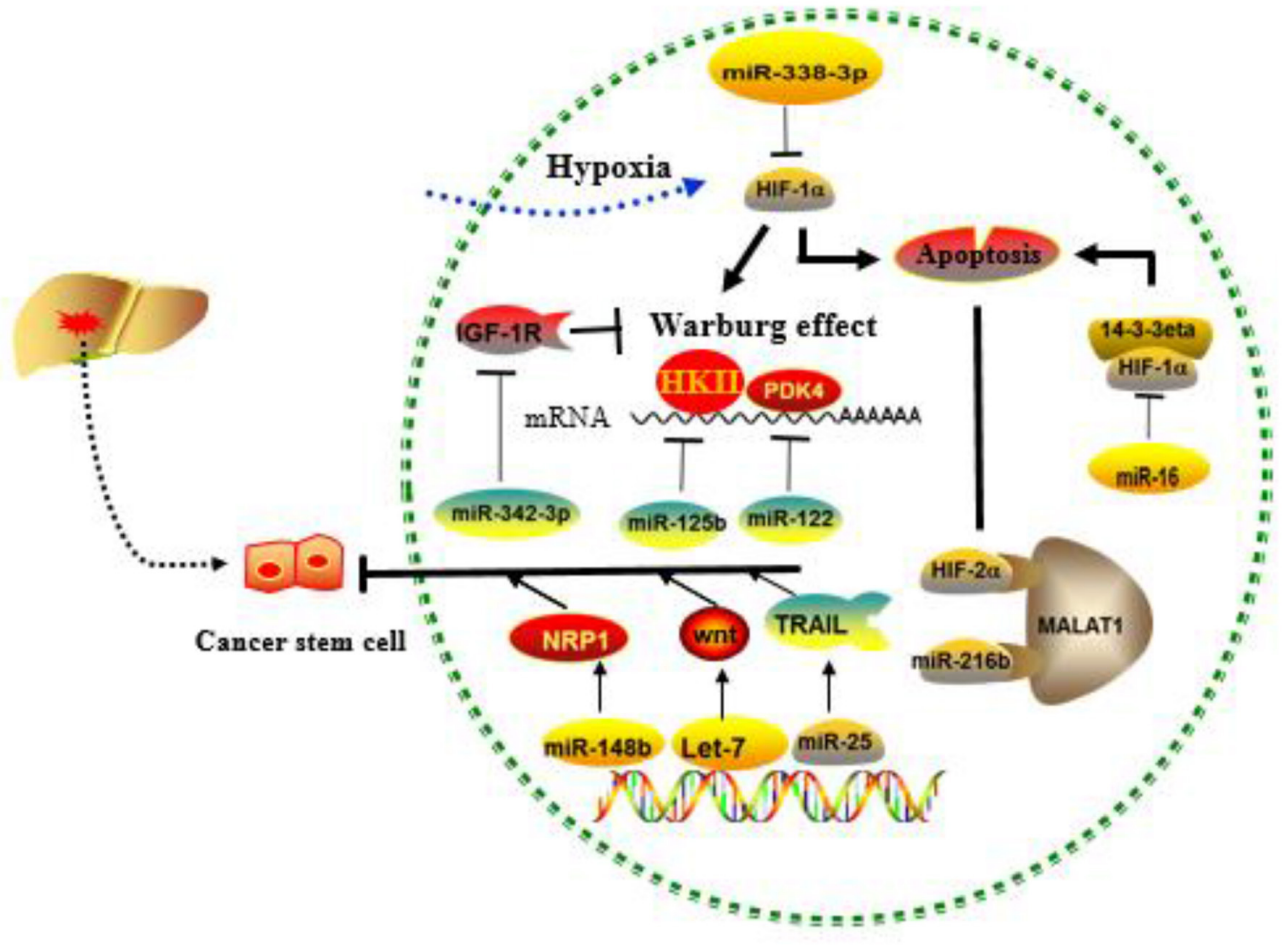
Role of cancer stem cells (CSCs), hypoxia, and the Warburg effect regulated by MICRORNAS (miRNAs) in the development of drug resistance. MiRNAs mainly affect drug resistance through negative regulation of key molecules in the above process.

Normal cells are often energized by the aerobic oxidation of sugar in the presence of sufficient oxygen and by the glycolysis pathway in the anoxic environment. However, most cancer cells do not obtain energy through mitochondrial oxidative phosphorylation even in the presence of sufficient oxygen, but instead, they use the aerobic glycolysis, a phenomenon defined as the Warburg effect (97). The Warburg effect converts glucose into lactic acid to produce ATP, and the accumulation of lactic acid in the extracellular environment will gradually increase, representing the transformation of oxidative phosphorylation to glycolysis. Indeed, increased consumption of glucose by cancer cells in order to produce energy is considered a major biochemical feature of the cancer cells (98). The intrinsic mechanism of the Warburg effect is mainly focused on the abnormalities of oncogenes and tumor suppressor genes, as well as related enzymes in sugar metabolism (99–101). miRNA-129-5p and miR-342-3p have been reported to affect HCC progression by blocking the Warburg effect (102, 103). MiR-125b, a tumor suppressor in HCC cells, downregulated in resistance of HCC cells to 5-FU, is likely mediated by miR-125b, a tumor suppressor in HCC cells that inhibit the target hexokinase II (HK II) related to glucose metabolism (104). Studies have also found that extracellular glucose and lactic acid are environmental factors for maintaining the characteristics in CSCs. Surprisingly, liver-specific miR-122 is down-expressed in CD133^+^ CSCs. When miR-122 is upregulated, PDK4 can be targeted to inhibit CSCs, which provides another solution for chemoresistance (105). MiR-342-3p inhibits IGF-1R-mediated PI3K/AKT/GLUT1 signaling pathway by reducing glucose uptake, lactate production, ATP production, and extracellular acidification rate (ECAR) and increasing oxygen consumption rate (OCR) of hepatoma cells to achieve the inhibition of cell proliferation (102). MiRNAs involved in tumor microenvironment-mediated resistance are summarized in Figure 4.

### Genes and Proteins That Regulate Apoptosis

The intrinsic or acquired resistance to apoptosis is one of the hallmarks of human cancers. Defects in apoptosis are the basis of drug resistance, and chemotherapy often fails (106). Aberrant expression or mutation of genes encoding crucial apoptotic proteins can induce cancer cells to have both the intrinsic survival advantage and inherent resistance to chemotherapeutic drugs. A large variety of genes regulating apoptosis have been discovered, such as the Bcl-2 family and tumor suppressor p53, p63/p73, PTEN, KRAS, and BRAF.

#### B-Cell Leukemia-2

Bcl-2 family is currently the most valuable gene family that regulates mitochondrial apoptosis. According to the different effects of Bcl-2 family on apoptosis, the Bcl-2 family genes can be divided into anti-apoptotic and pro-apoptotic. The anti-apoptotic genes include Bcl-2, Bcl-1, Mcl-1, Bag-4, Boo/Diva, and Bcl-w, whereas the pro-apoptotic genes include Bcl-xs, Bax, Bad, Bak, Hrk, and Bim (107, 108). The Bcl-2 family genes interact to determine cell fate. In the current review, we mainly focus on miRNAs involved in the anti-apoptotic signaling and drug resistance.

Bcl-2 is the most studied gene associated with anticancer drug resistance (109). It is confirmed that arsenic trioxide-induced apoptosis in HCC cells could be enhanced by overexpression of miR-539, and this has demonstrated to be mediated by decreased expression of Bcl-2, Bcl-xL, and phosphorylation of Stat3 (110). Overexpression of miR-34a has been shown to inhibit Bcl-2 and, therefore, promote sorafenib-induced apoptosis and toxicity in HCC cells (111). In another study, significantly reduced level of miR-363 has been found in cisplatin-resistant HepG2 (HepG2-R) cells as compared with the naïve HepG2 cells, but exogenous miR-363 remarkably has overcame cisplatin resistance in HepG2-R cells. Mechanistic studies have demonstrated that miR-363 directly targets Mcl-1 3′-UTRs, leading to decrease cisplatin resistance (112). LCSCs are regarded as the critical subset in the pathogenesis of HCC and therapy resistance. In addition, the activation of pro-survival pathways such as PI3K-AKT has been shown to promote drug resistance. Feng et al. have reported that the increased level of miR-25 is associated with the sensitivity of LCSCs to TRAIL-induced apoptosis (88). As such, knockdown of miR-25 may represent a potential strategy for increasing TRAIL by an induced killing effect targeting the PTEN/PI3K/AKT/Bad signaling pathway.

#### Phosphatase and Tensin Homolog Deleted on Chromosome 10

PTEN is a tumor suppressor gene that is essential for maintaining normal cell survival (113, 114) and is frequently mutated or deleted in several human cancers including HCC (115). Deletion of the PTEN gene or inactivation of a single PTEN allele can promote cancer development through negatively regulating AKT signaling (115). Hence, loss of PTEN leads to unrestricted proliferation of cancer cells and the loss of sensitivity of cancer cells to chemotherapeutic drugs (116, 117).

Recent studies have reported that miRNAs can regulate sorafenib resistance via interacting with the PTEN/AKT pathway (118). For example, it has recently been reported that miR-19a-3p induced sorafenib resistance by inhibiting PTEN and subsequent activation of PI3K/AKT, leading to increased EMT, cell migration, and acquisition of stem cell-like properties in HCC cells, rendering these cells more resistant to sorafenib (119). Similarly, inhibition of miR-205-5p can reverse the resistance of HCC cells to 5-Fu via activation of PTEN/JNK/ANXA3 axis (120). On the other hand, miR-760 has been reported to overcome resistance of HCC cells to Dox through activating PTEN-dependent PI3K/AKT signaling and targeting Notch1 pathway (121). These studies suggest that the mechanisms may differ among different drugs and cellular context.

#### Others

An important molecular switch that regulates proliferation, differentiation, and apoptosis is through activating MAPK and PI3K/AKT signaling pathways. The RAS family consists of KRAS, NRAS, and HRAS; among them, KRAS mutation is one of the most common molecular events, seen in 17–25% of human cancers (122). Tyrosine kinase inhibitors (TKIs) are representative drugs that function through blocking RAS pathways; hence, KRAS mutation is a mechanism leading to TKI resistance (123). Published studies have shown that miRNA-622 can inhibit KRAS signaling, leading to resensitization of sorafenib-resistant cells (124). Let-7g is reported to regulate EMT by downregulating the re-expressed KRAS (125). BRAF protein and KRAS are both upstream regulators in the RAS–RAF–MEK–ERK signaling pathway. In patients with advanced HCC, those with BRAF mutation are more aggressive and resistant to TKI (126). Another miRNA, miR-550a-3-5p, has also reported to reverse the resistance of HCC cells to BRAF inhibitors through direct targeting of YAP (127).

The miRNAs reported to modulate chemoresistance through intracellular mechanisms are summarized as Table 3. The miRNAs reported to modulate chemoresistance through extracellular mechanisms are summarized in Table 4.

**Table 3:** MiRNAs that modulate drug chemoresistance through intracellular mechanisms.

**MiRNA**	**Target**	**Mechanism**	**MiRNA regulated mechanism**	**Drug**	**References**
MiR-223	ABCB1	Membrane transporter	Negatively	Doxorubicin	(51)
MiR-27a	MDR1, β-catenin	Membrane transporter	Negatively	5-FU	(52)
MiR-133/326	ABCC1	Membrane transporter	Negatively	Doxorubicin	(57)
MiR-379	ABCC2	Membrane transporter	Negatively		(58)
MiR-338-3p	HIF-1α	Hypoxia	Negatively	Sorafenib	(94)
MiR-125b	HK II	Warburg effect	Negatively	5-FU	(104)
MiR-122	PDK4	CSC, Warburg effect	Negatively		(105)
MiR-342-3p	IGF-1R	Warburg effect	Negatively		(102)
MiR-539	Bcl-2, Bcl-xL, Stat3	Apoptosis	Negatively		(110)
MiR-34a	Bcl-2	Apoptosis	Negatively	Sorafenib	(111)
MiR-363	Mcl-1	Apoptosis	Negatively	Cisplatin	(112)
MiR-25	PTEN/PI3K/AKT/Bad	CSC, apoptosis	Negatively	TRAIL	(88)
MiR-19a-3p	PTEN/AKT	Apoptosis	Negatively	Sorafenib	(118)
MiR-216a/217	PTEN, Smad7	Apoptosis	Negatively	Sorafenib	(119)
MiR-205-5p	PTEN/JNK/ANXA3	Apoptosis	Negatively	5-FU	(120)
MiR-760	Notch1	Apoptosis	Negatively	Doxorubicin	(121)
MiR-622	KRAS	Apoptosis	Negatively	Sorafenib	(124)
Let-7g	KRAS	Apoptosis	Negatively		(125)

*miRNAs, microRNAs; 5-FU, 5-fluorouracil; CSC, cancer stem cell; PTEN, phosphatase and tensin homolog deleted on chromosome 10*.

**Table 4:** MiRNAs that modulate drug chemoresistance through extracellular mechanisms.

**MiRNA**	**Target**	**Mechanism**	**MiRNA regulated mechanism**	**Drug**	**References**
MiR-200c	E-cadherin, Zeb1/2	EMT	Negatively	Anti-EGFR	(69)
MiR-130a-3p	Smad4	EMT	Negatively	Gemcitabine	(70)
MiR-106a	PDGF-D, Twist1	EMT	Negatively	Gemcitabine	(71)
MiR-122	Zeb1/2, Snail1/2	EMT	Negatively		(72)
MiR-153	Snail	EMT	Negatively		(73)
Let-7a	Wnt	CSC	Negatively		(87)
MiR-25	PTEN/PI3K/AKT/Bad	CSC, apoptosis	Negatively	TRAIL	(88)
MiR-148b	NRP1	CSC	Negatively		(89)
MiR-125b	Smad2/4	CSC, EMT	Negatively		(90)

*EMT, epithelial–mesenchymal transition; CSC, cancer stem cell; PTEN, phosphatase and tensin homolog deleted on chromosome 10*.

**Figure 5:**
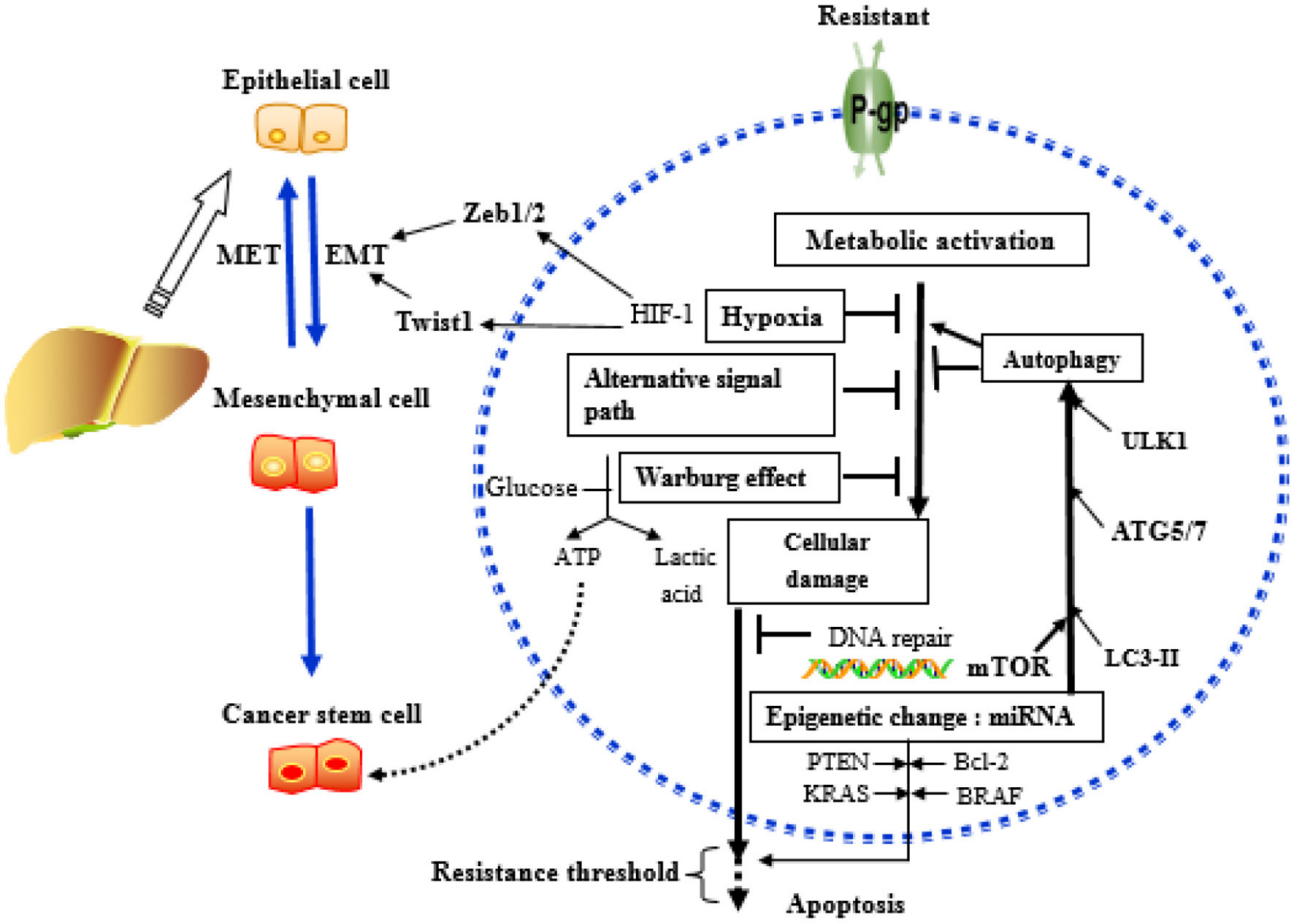
Key mechanisms related to drug resistance in hepatoma cells. MicroRNAs (miRNAs) can affect the outcome of chemotherapy, mainly acting on key molecules or signaling pathways related to the above mechanisms.

Figure 5 summarizes the key mechanisms involved in the development of drug resistance in HCC.

## Conclusions

Drug resistance is a major problem in cancer therapy. Understanding how drug resistance occurs and how it is regulated at the molecular level is a prerequisite for developing effective treatment approaches for liver cancer. At present, drug resistance is believed to be multifactorial involving abnormalities in autophagy, apoptosis, membrane transporters, EMT, and tumor microenvironment. The functions of drug resistance genes, the activation mechanism, and the regulation of miRNAs have been extensively studied. In this review, we present potential molecular, cellular, and microenvironmental mechanisms to understand drug resistance in HCC systematically and comprehensively. Autophagy can not only act as a housekeeper but also promote tumor cell death by enhancing the process of inducing apoptosis, playing a two-way regulation in the drug resistance process. P-gp and MRP are important components of the membrane transporter, which have a mediating effect on the entry and exit of chemotherapeutic drugs into tumor cells. EMT and MET, together with the critical signaling pathway, exist in the two transition processes, exerting a critical role in mediating tumor metastasis drug resistance. In addition, the biological processes involved in tumor microenvironment, such as CSCs, hypoxia, and the Warburg effect, function as an essential part in tumor progression, in which all miRNAs are involved in drug resistance. Likewise, Bcl-2, PTEN, KRAS, and BRAF are the genes that regulate apoptosis, acting as an important signaling molecule of their downstream pathway involved in the formation of tumor resistance. The resistance to tumors may be the result of synergistic and mediating effects of multiple genes and multiple mechanisms, but the detailed mechanism of MDR formation in tumor cells has not yet been fully elucidated. The review provides critical information on how miRNAs regulate the development of drug resistance in HCC and will shed light on the development of novel approaches for tackling drug resistance in liver cancer management.

## Author Contributions

FX selected the topic and directed writing. LQ reviewed it. YL wrote manuscript. QL collected information. All authors contributed to the article and approved the submitted version.

## Conflict of Interest

The authors declare that the research was conducted in the absence of any commercial or financial relationships that could be construed as a potential conflict of interest.
